# Context and Literality in Idiom Processing: Evidence from Self-Paced Reading

**DOI:** 10.1007/s10936-020-09719-2

**Published:** 2020-07-14

**Authors:** Sara D. Beck, Andrea Weber

**Affiliations:** University of Tübingen, Wilhelmstraße 50, 72074 Tübingen, Germany

**Keywords:** Idioms, Figurative language, Language processing, Context, Self-paced reading

## Abstract

In a self-paced reading study, we investigated how effects of biasing contexts in idiom processing interact with effects of idiom literality. Specifically, we tested if idioms with a high potential for literal interpretation (e.g., *break the ice*) are processed differently in figuratively and literally biasing contexts than idioms with a low potential (e.g., *lose one’s cool*). Participants read sentences that biased towards a figurative or literal reading of idioms and continued with resolutions that were congruent or incongruent with these biases (e.g., [The new schoolboy/the chilly Eskimo] just wanted to *break the ice* [with his peers/on the lake]…). While interpretations of high-literality idioms were strengthened by supporting contexts and showed costs for incongruent resolutions, low-literality idioms did not show this effect. Rather, interpreting low-literality idioms in a literal manner showed a cost regardless of context. We conclude that biasing contexts are used in a flexible process of real-time idiom processing and meaning constitution, but this effect is mediated by idiom literality.

## Introduction

Idioms challenge standard notions of meaning composition as they are, by definition, multi-word strings with a figurative meaning that differs from the sum of its parts. For example, the idiom *to take the bull by the horns* means, figuratively, to take charge of a difficult situation. However, a literal interpretation, denoting an event in which a male cow is grabbed by its horns, is also available following standard meaning composition. In recent years, a number of studies have shown that the availability of these two meanings can be influenced by linguistic context, and while there is a general consensus that context can facilitate access to the figurative meaning of an idiom (e.g., Fanari et al. 2010), there is less agreement on the impact of context on the availability of the literal interpretation (e.g., Rommers et al. 2013; Holsinger and Kaiser 2013). The current study adds to the body of literature examining contextual facilitation, by specifically considering the mediating effect of idiom literality.

Idiom literality refers to the variation between idioms in their potential to be interpreted literally. While *to take the bull by the horns* can be used figuratively just as well as literally (i.e. has high literality), an idiom like *to be on cloud nine* has a low potential for literal interpretation in the absence of any contextual information (i.e. has low literality). Figuratively, it means to be very happy, and literally, a context is needed in order to create a situation in which a person might find themselves “on” a cloud called “nine.” This variance in literality could mediate contextual effects on literal and figurative interpretations and particularly help to explain the varying effects of context on literal interpretations in previous studies. For high-literality idioms, supporting contexts could strengthen both the figurative and the literal interpretation, since both interpretations have a high potential to start with. For low-literality idioms, supporting contexts could still strengthen the figurative interpretation, but fail to affect the literal interpretation.

We tested our assumptions in a self-paced reading study in English with idioms of varying literality. Following up on the methods used in a self-paced reading study conducted on phrasal verbs (Holsinger and Kaiser 2013), our study examined idioms which were embedded into sentences containing literally- or figuratively-biasing contexts and followed by resolutions that were congruent or incongruent with the bias (e.g., [The new schoolboy/the chilly Eskimo] just wanted to *break the ice* [with his peers/on the lake]…). By comparing the costs and benefits of processing these expected and unexpected variations in sentences containing idioms of varying literality, our study looked closely at how literal and figurative meanings are integrated into sentential meaning in online processing.

While our study does not directly intend to test individual models of idiom processing, we will first outline the various psycholinguistic models of idiom processing in order to place it within this field of research before we discuss the relevant literature for the contributing factors: context and literality.

### Idiom Processing

Idiomatic models of processing have offered various accounts of the duality of meaning presented by idioms, and the role of literal meaning in idiom processing varies starkly among these models. Early models treated idioms as complex words for which the figurative meaning is directly retrieved from the lexicon. The consequence of such models is that literal meaning composition plays no direct role in accessing the figurative meaning of an idiom. The order of this composition and retrieval approach could be considered as either literal-first, as in the Lexical Access model (Bobrow and Bell 1973); figurative-first, as in the Direct Access Hypothesis (Gibbs 1980); or parallel in both literal composition and figurative retrieval, as in the Lexical Representation hypothesis (e.g., Swinney and Cutler 1979). Of these models, only the Direct Access Hypothesis implies that literal composition need not occur. According to this hypothesis, idiomatic retrieval can begin immediately without considering literal composition. In the other two cases, literal composition occurs obligatorily, though the order, and thus predictions for speed of access, differs.

The stepwise nature of meaning access implied by such lexical theories of idiom retrieval, however, is not substantiated by more recent psycholinguistic research. Figurative meaning has consistently been shown to be available online (e.g., Beck and Weber 2016a; Cacciari and Tabossi 1988; Libben and Titone 2008) and sometimes even earlier than literal meaning (e.g., Cacciari and Corradini 2015), but access to one interpretation does not necessarily preclude access to the other. Cacciari and Tabossi (1988) and, later, Beck and Weber (2016a) demonstrated that, in cross-modal priming experiments, the activation of individual literal constituents occurs quickly and in addition to the activation of figurative meaning when presented in non-biasing sentence contexts (e.g., *kick the bucket* actives DIE and PAIL). Though few studies have looked at both literal and figurative interpretations on a phrasal level, particularly without the impact of biasing context, Ortony et al. (1978) found that literal interpretations were faster than figurative ones where only short, non-biasing, contexts were present, while longer contexts showed the reverse. Using conventional metaphors, McElree and Nordlie (1999), found no differences in the timing for access to both interpretations using a speed-accuracy trade-off procedure, providing even more evidence against simple stepwise models of idiomatic processing.

An alternative to such models can be found in so-called hybrid approaches (e.g., Cacciari and Tabossi 1988; Cutting and Bock 1997; Sprenger et al. 2006). These hybrid models attempt to integrate the processing of idioms into the framework of existing processing and representational accounts. The Configuration Hypothesis (Cacciari and Tabossi 1988), for example, assumes that literal composition occurs necessarily until enough information has accrued for the idiom to be recognized as such. Thus, processing is literal by default until signaled to proceed otherwise, and the literal meaning plays no clear role after recognition. Libben and Titone (2008) added to this idea in their Constraint-based Model of Idiom Processing by suggesting that idiomatic properties, such as compositionality or literality, as well as linguistic context can affect access to meanings at different times during processing (see also Titone and Libben 2014), and access to figurative meaning also appears to accrue cumulatively.

The Hybrid Model of Idiom Representation, a model of idiom production put forward by Sprenger et al. (2006), links the meaning of literal constituents more clearly to the figurative meaning of an idiom on a representational level (see also Cutting and Bock 1997). Individual lemmas and *superlemmas*, or representations of idioms on the lexical-syntactic level, are simultaneously activated starting with the first word of the idiom, unlike in the Configuration Hypothesis. Activation of both the literal and figurative meanings compete with one another as a sentence unfolds, and access to the figurative meaning follows a spreading procedure that increases as more information becomes available, much like the literal composition process. As in the Configuration Hypothesis, a clear prediction for literal composition after recognition is not made, though the employment of meaning competition, as opposed to a building recognition in the Configuration Hypothesis and Constraint-based Model, suggests that literal composition may continue on some level. However, in all of these hybrid models, access to the figurative meaning proceeds cumulatively, either via building recognition in the case of the former or increasing semantic activation in the case of the latter, and this process has the potential to be impacted by local and global factors such as literality and context in both cases.

### Contextual Effects

A number of studies on preceding linguistic context provide evidence that figuratively biasing contexts can ease access to figurative meaning in idioms (e.g., Colombo 1993; Fanari et al. 2010; Holsinger and Kaiser 2013; Ortony et al. 1978). Studies using preceding linguistic biasing contexts have found faster paraphrasing of figurative interpretations (e.g., Gibbs 1980), better monitoring of matching, rhyming, and related words (e.g., Estill and Kemper 1982), larger priming effects in cross-modal priming tasks (e.g., Colombo 1993), as well as faster reading times (e.g., Colombo 1993, 1998). Some studies have even suggested that figuratively biasing contexts might be a necessary condition for early activation of figurative meanings, particularly in the case of unpredictable or short idioms, and idioms with high literality (e.g., Cacciari et al. 2007; Colombo 1993; Fanari et al. 2010).

Access to the literal meaning of individual constituent words or phrasal meaning has not been shown to be as consistently affected by context in psycholinguistic studies. While cross-modal priming studies have shown literal constituent activation in idioms in neutral contexts (e.g., Beck and Weber 2016a; Cacciari and Tabossi 1988), EEG evidence found in a study by Rommers et al. (2013) showed that the literal meaning of final words in predictable idioms (e.g., *lamp* in *liep tegen de lamp*, ‘walked against the lamp’, meaning to get caught) was not activated when the words were presented in idiomatic sentences. When the same final words were presented in literal sentences (e.g., *draaide het peertje in de lamp*, ‘screwed the light bulb into the lamp’), their literal word meaning was activated. That is, an N400 effect for semantically related words (e.g., *kaars*, ‘candle’) was found in literal sentences, but no N400 effect was found in idiomatic sentences, a result suggesting that literal interpretation was rendered unnecessary in this context. In another EEG study, Canal et al. (2017) similarly found evidence suggesting facilitation for figurative and literal meanings following biasing contexts, this time for the entire phrase rather than only constituent words. In line with Rommers et al. (2013), evidence for qualitative changes in processing after idiom recognition suggests that literal composition mechanisms are suppressed or discontinued following a figurative context. Thus, context has an impact on both interpretations during processing.

Alternatively, there is also some evidence that the literal meaning of idioms is not improved by preceding contexts to the same extent that figurative bias improves access to idiomatic interpretations, if at all (e.g., Gibbs 1980). Like idioms, phrasal verbs can have a literal and figurative interpretation, and Holsinger and Kaiser (2013) investigated the costs for recovery of meaning in phrasal verbs where the resolution of the verb was either congruent or incongruent with expected literal or figurative interpretations (e.g., The daring archaeologist/The hungry waitress *dug into* the tomb/the sandwich). Their self-paced reading study showed that figurative interpretations benefitted from the presence of biasing contexts, but literal interpretations did not show differences regardless of whether the biasing context was figurative or literal. Furthermore, they found that the greatest processing costs occurred when a figurative interpretation followed a literal biasing context. Thus, unlike the previous studies discussed above (Canal et al. 2017; Rommers et al. 2013), Holsinger and Kaiser conclude that literal composition occurs necessarily, regardless of context, whereas context is required for achieving early access to figurative meaning. Notably, while the reading study by Holsinger and Kaiser used phrasal verbs rather than idioms and a mismatch design focusing on meaning integration (i.e., biasing contexts matched or mismatched with a later resolution in the sentences), the EEG studies already mentioned compared the effects of contexts in figurative sentences with idioms to comparable literal sentence processing (Canal et al. 2017; Rommers et al. 2013). These key differences leave unanswered questions about the impact of context on literal interpretations and the obligatory nature of literal composition in idiom processing.

### Idiom Literality

Idioms vary starkly in their potential to be used in a literal sense (e.g., Popiel and McRae 1988), and this potential for literal interpretation, referred to here as literality, has been shown to impact idiom processing. Literality is commonly based on ratings across a scale determining the plausibility of a literal interpretation of an idiom (see e.g., Beck and Weber 2016a; Titone and Connine 1994a, b). This property is closely related to other idiomatic properties such as the ambiguity of an idiom’s meaning and is sometimes even referred to interchangeably so (see e.g., Briner and Virtue 2014; Cacciari and Tabossi 1988). This overlap in definitions between such properties may be because, in ambiguous idioms, the likelihood of figurative and literal interpretations is comparable, as may be the case in some idioms with a high literality. The current study will focus on literality, as defined above, and target idioms at both ends of the scale: high- or low-literality idioms.

While it might seem feasible that the potential for both literal and figurative interpretations in the meaning of high-literality idioms (e.g., *to take the bull by the horns*) slows down processing, several studies have found the opposite pattern. Cronk and Schweigert (1992), for example, observed faster reading times for high-literality idioms, and Mueller and Gibbs (1987) found that high-literality idioms were classified more quickly than low-literality idioms. Both attributed these findings to multiple entries in the lexicon (i.e. one figurative and one literal), thereby increasing the likelihood of encountering the phrase in either usage and contributing to overall increased speed in meaning access.

Literality has also been found to interact with other idiomatic properties to affect access to figurative and literal meaning. Titone and Connine (1994b), for example, showed that literality affects access to literal constituent meaning in highly predictable idioms (i.e., idioms in which the final word can be predicted). In their cross-modal priming study, highly predictable, low-literality idioms in neutral contexts showed no activation of words semantically related to the final constituent words (e.g., *burn the midnight oil* and FUEL). However, for unpredictable idioms, literal constituent meaning was activated in both high- and low-literality idioms. Titone and Connine interpreted the results to mean that competition between figurative and literal meanings may stay active when relevant, for instance in high-literality idioms, but this competition can decay where not necessary (see also e.g., Giora 1997: Graded Saliency Hypothesis).

What is lacking in the current state of research, however, is a look at context and literality together, in particular, an investigation of how context and literality might affect one another. For instance, in unpredictable idioms, where Titone and Conine (1994b) found literal activation regardless of literality, it is unclear whether the presence of biasing contexts might impact this literal activation. A re-examination of both high- and low-literality idioms in the presence of context—both literal and figurative—may allow for a more precise picture of both the impact of literality on processing as well as the possible limitations of contextual effects where literality varies.

### Self-Paced Reading Experiment

To this end, we conducted a self-paced reading study in English to further determine the activation and role of literal and figurative meaning during the time-course of idiomatic processing. Like Holsinger and Kaiser (2013), we aimed to determine how context can impact access to these meanings in both cases where contextual expectations are met and turn out to be false. Specifically, we intended to focus on the point in reading at which either the confirmation of congruent meanings or the recovery from incongruent expectations occurs. We tested both high-literality and low-literality idioms in speakers of American English to investigate whether the effects of context vary with the literality of an idiom.

Our study had a total of three manipulations: idiom type (high- or low-literality idioms), biasing context (literal or figurative bias), and resolution (literal or figurative resolution). High-literality idioms, like *break the ice* (meaning figuratively to ease the nervousness in a social situation), followed contexts that either biased a literal or a figurative interpretation (e.g., literal: The cold Eskimo, who was eager to catch some fish; figurative: The new schoolboy, who didn’t know anyone in his class) and preceded resolutions that either corresponded to a literal or a figurative interpretation (e.g., literal: on the lake…; figurative: with his peers…). Low-literality idioms like *lose one’s cool* (meaning figuratively to lose control of one’s emotions) also followed the same type of biasing contexts (e.g., literal: The freelance writer, who often started political debates; figurative: The sweaty runner, who was recovering under a tree) and preceded corresponding resolutions (e.g., literal: from the shade; figurative: out of anger).

We predicted that our results for figurative interpretations should replicate those found by Holsinger and Kaiser (2013). Namely, there should be a benefit of faster reading times when a context biases for a figurative interpretation and a figurative resolution occurs compared to when a context biases for a literal interpretation and a figurative resolution occurs. These predictions are also in line with other contextual studies showing the benefits of figurative contexts (e.g., Colombo 1993). Since both high- and low-literality idioms have a high potential for a figurative interpretation (i.e., per definition all idioms have a figurative meaning), we predicted the facilitatory effect of figurative contexts to occur for both idiom types. For literal interpretations, however, we expected a difference in context effects for high- and low-literality idioms. Contrasting Holsinger and Kaiser (2013), we expected a benefit when a context biases for a literal interpretation and a literal resolution occurs compared to when a context biases for a figurative interpretation and a literal resolution occurs. Although neither Rommers et al. (2013) nor Canal et al. (2017) used a mismatch design, such a result would be in line with their studies as it suggests an influence of context also on the literal interpretation. If idiom literality is a mediating factor, then, in contrast with high-literality idioms, we would expect no benefit of a biasing context for low-literality idioms in the present study. This prediction would be in line with evidence from Titone and Connine (1994b) showing that literal activation may not occur in some cases for low-literality idioms, something which may be attributed to a lack of saliency for the literal meaning in these idioms (see e.g., Giora 1997). If literality, however, does not outweigh the general saliency of idioms’ figurative interpretations, differences in contextual influence on literal interpretations may not be present or measurable in such an experiment.

## Method

Participants.Fifty-two native speakers of American English (35 female; average age of 21.62, SD = 2.44) received financial compensation for taking part in the study. Participants were recruited at the University of Maryland from the Department of Linguistics subject pool and grew up in monolingual households. A total of 6 participants were left-handed, and all participants had normal hearing and normal to corrected vision.

Materials.The experiment consisted of 22 target and 78 filler trials. Sentences in target trials began with a biasing context (noun phrase + relative clause) followed by the infinitive form of the idiom, followed by a resolution (prepositional phrase) congruent or incongruent with contextual expectations, and ended with two additional short phrases shared across all conditions (see Appendix). Two example stimuli with all four conditions are provided in Table 1. As the experiment was conducted using a phrase-by-phrase reading paradigm, each column in the table represents the phrases seen by participants during the experiment. Phrases were controlled for letter length and average word frequencies, with only minimal differences between conditions (see analysis section for more information). All idioms had the same syntactic structure (to-infinitive verb + determiner + noun) and were short and unpredictable.

**Table 1:** Example stimuli

	Biasing Subject	Biasing context 1	Biasing context 2	Pre-idiom	Idiom	Resolution	Resolution + 1	Wrap-up
*High literality*								
a.	The new schoolboy	who didn’t know	anyone in his class	just wanted to	break the ice	with his peers	sooner than later	monday morning
b.	The new schoolboy	who didn’t know	anyone in his class	just wanted to	break the ice	on the lake	sooner than later	monday morning
c.	The chilly Eskimo	who was eager	to catch some fish	just wanted to	break the ice	on the lake	sooner than later	monday morning
d.	The chilly Eskimo	who was eager	to catch some fish	just wanted to	break the ice	with his peers	sooner than later	monday morning
*low literality*								
a.	The emotional writer	who often started	political debates	didn’t want to	lose his cool	in his anger	too quickly	that morning
b.	The emotional writer	who often started	political debates	didn’t want to	lose his cool	from the shade	too quickly	that morning
c.	The overheated runner	who was resting	under a tree	didn’t want to	lose his cool	from the shade	too quickly	that morning
d.	The overheated runner	who was resting	under a tree	didn’t want to	lose his cool	in his anger	too quickly	that morning.

Sixty-eight sentences varying in length and structure were selected as filler trials, and ten of these sentences also contained idioms. Additionally, ten filler trials consisted of basic arithmetic questions (e.g., (30 − 28)*5 = ?). One third of all trials, including all target trials, were followed by comprehension questions targeting differing types of sentential information. The comprehension and arithmetic questions were included to hold participants’ attention and ensure concentration throughout the task, and correctness of response was considered in the analysis. All experimental items were divided into 4 lists, counterbalanced so that each idiom occurred only once per list, but idiom-type and context- and ending-conditions were balanced between the lists. In order to do so with an uneven number of experimental items, the lists varied slightly by resolution-type in the ratio of congruent and incongruent endings (half of the lists had one additional congruent ending and half one additional incongruent ending), but were equivalent in all other aspects. The order was then randomized and reversed for each list so that there were 8 lists in total. All lists contained the same filler items.

Three regions of interest, also labeled in Table 1, were identified for our analysis: the Idiom, the Resolution, and the Resolution + 1 (the phrase following the resolution) regions. However, our main predictions concern the Resolution +1 region. Since previous studies have found that meaning is typically available later in short and unpredictable idioms than in longer, more predictable ones (e.g., Titone and Connine 1994a, b), we expected the effects of (mis-)matching contexts to show up in the region following the resolution rather than during the resolution itself. These expectations align with the late effects found by Holsinger and Kaiser (2013). Furthermore, because the Resolution + 1 region is identical across conditions for each item (see Table 1), observed differences in reading times based on context and resolution cannot be attributed to differences in lexical items or syntactic structure in this region.

Before deciding on the 22 target idioms, a total of 30 idioms were pre-selected from the 300 idioms in the English-German Database of Idiom Norms (Beck and Weber 2016b) based on a number of factors that are known to affect idiomatic processing (see e.g., Titone and Libben 2014). Only unpredictable idioms (i.e. with a low predictability score) with a high subjective familiarity were pre-selected (ratings higher than 3.5 on a 7-point scale). Idioms shared a VP syntactic structure, beginning with a verb and ending with a noun (e.g., *break the ice*), but they differed in levels of literality (high literality had a minimum of 4 and low-literality idioms had a maximum of 3 on a 7-point scale). For all 30 pre-selected idioms, biasing sentences were constructed and subjected to norming studies.

*Norming* Two norming studies were conducted on the pre-selected 30 idioms and their biasing sentences in order to choose the items that were a) most appropriately biased for a literal or figurative reading of the idioms following the given contexts and b) most plausible (see e.g., Ratcliff 1987). Based on the results from these norming studies (see below), a total of 22 idioms were selected for the reading study: 11 high-literality idioms (average of 5.0) and 11 low-literality idioms (average of 2.8; *t* = 14.12, *p* < 0.001). The selected 22 idioms had mean constituent frequencies of 3.25 per million and were rated as highly familiar by L1 speakers (average of 6.5).

*Norming Study 1: Strength of Context* In a multiple-choice test, 40 American English participants were presented with the idioms preceded by either their figuratively or literally biasing contexts and were asked to choose the meaning of the highlighted phrase (the idiom) that most appropriately fits the provided sentential context. Each participant saw only one biasing context for each idiom, and four answer choices were available: the literal meaning, the correct figurative meaning, an incorrect figurative meaning, and “I don’t know”.

For the final selection of 11 high literality idioms, the percentages of completions consistent with the biasing context were 98% for figurative contexts and 52% for literal contexts respectively; for the final selection of 11 low literality idioms, the percentages of consistent completions were 97% for figurative contexts and 30% for literal contexts respectively. Linear models were conducted on the average correct completions based on context using *idiom literality* (high- and low-literality) and *biasing context* (figurative and literal) as fixed effects. Our model confirmed that figurative contexts were completed consistently more often than literal contexts (*β *= − 20.4, *t *= − 2.8, *p *< .01) and that for literal contexts, the high-literality idioms were completed consistently more often than low-literality idioms (*β *= − 46.4, *t *= − 8.8, *p *< .001). Thus, in a number of cases participants responded with figurative interpretations even when a literal context rendered them inappropriate, thereby in fact ignoring contextual information. If this preference for figurative interpretations in the norming study indeed implies that figurative contexts bias more than literal contexts, it is possible that we will not see effects of literal bias in our self-paced reading results. Nonetheless, in order to best capture the potential individual variation based on item, biasing strength, included as the by-item average of correct completions based on context, was later included as a predictor in the regression models in our analysis of reading times where warranted.

*Norming Study 2: Plausibility* Eighty American English participants were asked to rate the 30 pre-selected idioms with their four biasing sentence contexts (see Table 1) on how plausible the situation described by the sentences was (on a 7-point scale). Each participant only saw each idiom in one of the four conditions.

For the final selection of 22 items, the mean plausibility rating was 4.15 (SD= 1.17), and item averages ranged from 2.16 to 6.85, suggesting that while no items were rated as completely implausible, some were rated as very plausible. We again used simple linear models to verify any differences between items based on our predictive variables. Using the mean plausibility rating as the dependent factor, *idiom literality* (high- and low-literality), *biasing context* (figurative and literal), and *resolution type* (figurative and literal) were included as fixed effects. Our model showed that ratings for literal endings were overall lower than those for figurative endings (*β *= − 1.9, *t *= − 5.8, *p *< .001), and literal contexts were likewise rated lower than figurative contexts (*β *= − 2.2, *t *= − 6.7, *p *< .001). Additionally, an interaction of resolution by context suggested that congruent endings (literal contexts and resolutions/figurative contexts and resolutions) had higher ratings than incongruent resolutions (*β *= 3.5, *t *= − 7.5, *p *< .001), and a three-way interaction suggests that low-literality idioms were rated worse than high-literality idioms in the condition with a literal bias and resolution (*β *= − 1.6, *t *= − 2.2, *p *< .05), whereas low-literality idioms showed higher plausibility ratings in cases where endings were congruent with their contexts.

A further look at the comments section at the end of the ratings study suggested that participants were likely responding to preferential use of idioms rather than the plausibility of the sentences as described in the instructions, and it is possible that this norming study did not fully capture plausibility as intended. The differences between both congruent and incongruent endings as well as the preference for low-literality idioms with a figurative resolution also aligns with these expectations, as both cases represent unlikely uses of idioms. In spite of the difficulties in capturing this effect, in order to account for possible item differences based on plausibility, as collected in this norming study, the average plausibility ratings were included as a predictor in the regression models of the reading data where justified.

*Procedure* Participants gave informed consent following the ethical guidelines of The University of Maryland College Park and were tested individually in quiet rooms at the Language Science Center. The experiment was programmed and executed using E-Prime (Psychology Software Tools 2013), and a subsequent idiom recognition test (described below) and a language background questionnaire were completed using Adobe Acrobat. Participants wore noise-cancelling headphones to ensure that no noise distractions occurred during the reading study, and responses were recorded with the spacebar of a keyboard.

Participants began the reading study with four practice trials, and halfway through the experiment a self-timed break was offered. A standard moving-window phrase-by-phrase presentation was used in which each phrase was masked by hyphens corresponding in length to the phrase to be presented. Phrase-by-phrase rather than word-by-word was used in order to better mimic natural reading patterns and avoid a forced incremental processing pattern (e.g., Jegerski 2014), which might directly affect the questions at hand. This method, while not precise enough to measure fine differences in constituent meaning activation as the sentence unfolds, provides a good balance between natural reading and experimental procedures that can capture the costs and benefits of integrating activated meanings into contexts. Participants were instructed to begin a trial by pressing the space bar, and to press the space bar again once they had read the phrase presented. After pressing the space bar, the phrase again became masked, and the new phrase was unmasked. 30 of the 90 reading trials were followed by multiple choice comprehension questions, and 10 by basic arithmetic problems. After the reading study, participants completed an idiom recognition test and a language background questionnaire. In the idiom recognition test, participants were presented with all 22 target idioms in the sentences from the condition including the figurative context and figurative resolution. The task was to select the figurative meaning of the idiom from a randomly ordered multiple choice selection including (1) the figurative meaning, (2) a possible literal meaning, (3) an incorrect meaning, and (4)“I don’t know.” if the idiom was unfamiliar. This task helped ensure that the idiomatic meaning was indeed familiar for the participants of the study. The results of the recognition task were used to inform the analysis of our data.

## Results

We used R (R Core Team 2017) and lme4 (Bates et al. 2015a, b) to perform linear mixed effects analyses of the relationships between *biasing context* (figurative, literal), *resolution type* (figurative, literal), and *idiom literality* (high-literality, low-literality) on normalized reaction times in each of the three previously identified areas of interest: *idiom*, *resolution*, and *resolution *+* 1* regions. Trials were only included in the analyses if answers to the directly following comprehension questions were answered correctly. One participant fell below a threshold of answering 85% of questions correctly (68% correct) and was therefore excluded from the analysis. Two idioms (*play the field* and *turn the tide*) were also excluded as the idiom recognition test following the reading study revealed that more than half of participants were not familiar with these idioms. Outliers were excluded based on a visual inspection of reading times in each section. First, theoretically irrelevant times were excluded, second, inspection of the mean and general distribution of overall reading times were used to determine critical reading times for each region on an individual basis. Critical reading times were identified as 150–1500 ms in the *idiom* section, 150–1800 ms in the *resolution* section, and 150–1500 ms in the *resolution *+ *1* section, and 3.89%, 2.62%, and 4.85% of the data was excluded in each region respectively. The difference in size between regions is in line with the mean length, difficulty, and average reading times in each region. Table 2 reports the mean and log-transformed reading times for the phrases in each region across all factors.

**Table 2:** Raw reading times (ms and log) by region and main effects

Region	Resolution	Figurative context				Literal context			
		High-literality		Low-literality		High-literality		Low-literality	
		RTs	log RTs	RTs	log RTs	RTs	log RTs	RTs	log RTs
Idiom	Figurative	607.62	6.33	634.95	6.37	611.40	6.34	647.32	6.40
	Literal	598.56	6.33	611.56	6.36	607.57	6.34	653.87	6.40
Resolution	Figurative	639.59	6.36	718.83	6.42	680.36	6.48	756.30	6.53
	Literal	617.58	6.33	675.48	6.37	641.68	6.43	711.87	6.47
Resolution + 1	Figurative	572.19	6.26	613.32	6.34	616.95	6.36	615.57	6.36
	Literal	610.36	6.34	705.41	6.48	605.24	6.31	680.60	6.43

The analyses for each region of interest were done separately on normalized log-transformed values (see Baayen 2008) following the same procedure each time. With the exception of the idiom section (see details below), independent measures and fixed factors, numerically centered around 0, were coded and included as follows: *biasing context* (figurative: 0.5, literal:  − 0.5), *resolution type* (figurative: 0.5, literal:  − 0.5), and *idiom literality* (high-literality: 0.5, low-literality:  − 0.5). All three theoretically relevant factors were kept in the models, and items and participants were included as random factors with random slopes. A maximally justified random effects structure was determined for each region by stepwise selection and model comparison (see e.g., Bates et al. 2015a, b) consulting RePsychLing (Baayen et al. 2015). To account for additional variation, fixed factors of *trial order*, *region length* (in number of letters), *average lexical frequency*, and norming values for *idiom familiarity*, *plausibility,* and *strength of context* were also included in full models, and were eliminated from the models if backward stepwise selection showed that they did not improve the model. While these additional factors did not contribute to the interpretation of the results, their inclusion in the final model was justified by model comparisons, and any additional effects found were more likely to be caused by our manipulations rather than these factors. After models were selected, extreme outliers with a distance of greater than 2.5 standard deviations from zero were removed in order to improve model fit (see e.g., Baayen 2008), this process excluded an additional 1.72%, 1.97%, and 2.40% of the data respectively. The analyses for each section are discussed in the following, with a main focus on the *resolution *+* 1* region as it is the only region which directly compares reading times of the same phrases in all four conditions, and it is a likely point in time for effects of the matching or mismatching resolutions to be observable.

Reading Times in the Idiom Region.The model for reading times for the idiom region did not include *resolution type* or the norming factor for *plausibility* as the ending has not yet been encountered at this point. The final model output is displayed in Table 3. Significant effects of *region length* (*β *= .038, *t *= 2.36, *p* < .05) and *trial order* (*β *= − .06, *t *= − 7.08, *p *< 0.001) suggest that idioms with more letters took longer to read and that reading times improved over the course of the experiment. These effects are consistent throughout the successive regions but did not contribute to the overall findings other than to justify their inclusion in the models and will therefore be listed in the tables for models but not discussed further in the text.

**Table 3:** Idiom region LMER model output

Fixed effects	*β* (SE)	*t*	*p*
Intercept	6.368 (.045)	140.324	2.00E−16***
Biasing context	− 0.005 (.022)	− 0.256	0.8016
Idiom literality	− 0.051 (.033)	− 1.549	0.1398
Region length	0.038 (.016)	2.361	0.0309*
Trial order	− 0.067 (.009)	− 7.088	3.74E−10***
Context × Literality	0.037 (.043)	0.865	0.4009

Random effects	Variance	SD
Subject	0.075	0.274
Biasing context	0.002	0.039
Item	0.004	0.060
Biasing context	0.002	0.048

*p < .05; **p < .01; ***p < .001

**Fig. 1:**
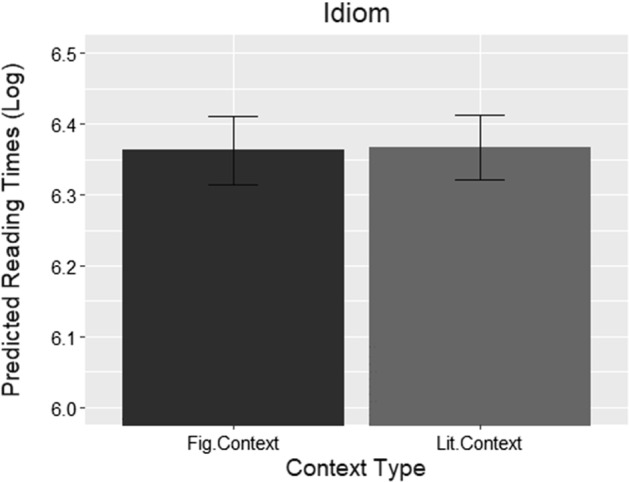
Log reading times predicted by the final model at the idiom region collapsed across literality. The black bar represents mean log reading times for idioms following a figurative context and the grey bar following a literal context, both with standard error bars

The predicted log reading times for the idiom region, collapsed across literality, can be seen in Fig. 1. All predicted values and standard error bar values were obtained from the R package ggeffects (Lüdecke 2018). As reflected in the graph, no significant interactions between factors emerged, and there was no significant effect of context at this point in reading.

Reading Times in the Resolution Region. For the resolution region, Table 4 displays the output of the best-fitting model after backward, stepwise selection and further removal of extreme residuals. Again, *region length* and, marginally, *trial order* effects were significant. Additionally, *idiom familiarity* improved overall reading times in this region (*β *= -.04, *t *= − 2.26, *p *< 0.05), suggesting that reading times improve with increasing familiarity. *Resolution type* was also significant as a main effect (*β *= -.04, *t *= 1.96, *p *< 0.05) and as an interaction with *biasing context* (*β *= -.09, *t *= − 2.00, *p *< 0.05).

**Table 4:** Resolution region LMER model output

Factor	*β* (SE)	*t*	*p*
Intercept	6.409 (.046)	137.713	2.00E−16***
Biasing context	− 0.028 (.028)	− 0.995	0.3341
Idiom literality	− 0.066 (.039)	− 1.667	0.1144
Resolution type	0.043 (.022)	1.969	0.0494*
Region length	0.030 (.016)	1.852	0.0827.
Trial order	− 0.089 (.011)	− 7.828	1.98E−14***
Idiom familiarity	− 0.043 (.019)	− 2.26	0.0375*
Context × literality	− 0.030 (.056)	− 0.532	0.6022
Context × resolution-type	− 0.090 (.045)	− 2.004	0.0455*
Literality × resolution-type	0.015 (.045)	0.353	0.7243
Context × literality × resolution-type	− 0.018 (.089)	− 0.209	0.8347

Random effects	Variance	SD
Subject	0.080	0.282
Item	0.003	0.053
Biasing context	0.006	0.077

.*p *< 0.1; *p < .05; **p < .01; ***p < .001

**Fig. 2:**
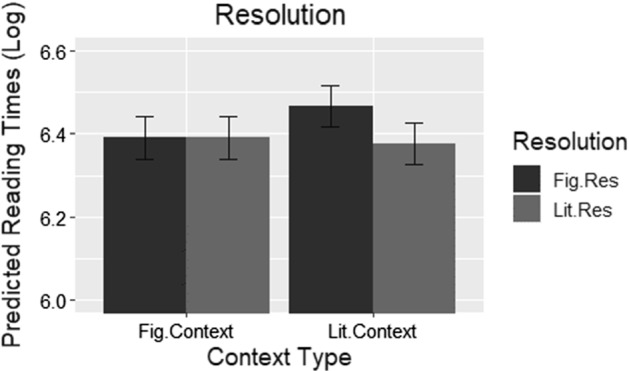
Log reading times predicted by the final model at the resolution region collapsed across literality. The black bars represent mean log reading times resolving figuratively (Fig.Res) and the grey bars resolving literally (Lit.Res), all with standard error bars

In order to better interpret this interaction, a post hoc analysis was conducted using simple slopes (e.g., Aiken and West 1991). By recoding *biasing context* (first as figurative context = 0, literal context = 1, subsequently reversed), its effect on *resolution type* can be examined more clearly. The same model used in the original analysis was run using the change in coding listed above, and the effect of *resolution type* is significant when contexts are literal (*β *= .08, *t *= 2.78, *p *< 0.01) and not figurative (*β *= .00, *t *= − 0.03, *p *= 0.96). Namely, as displayed in Fig. 2, where contexts are literal, reading times are significantly slower for figurative resolutions. While this effect is in line with one of the main conclusions of Holsinger and Kaiser (2013), it should be noted that the comparisons in this region were between different lexical units, and this result will be placed in this context in the discussion section.

### Resolution + 1 Region

**Fig. 3:**
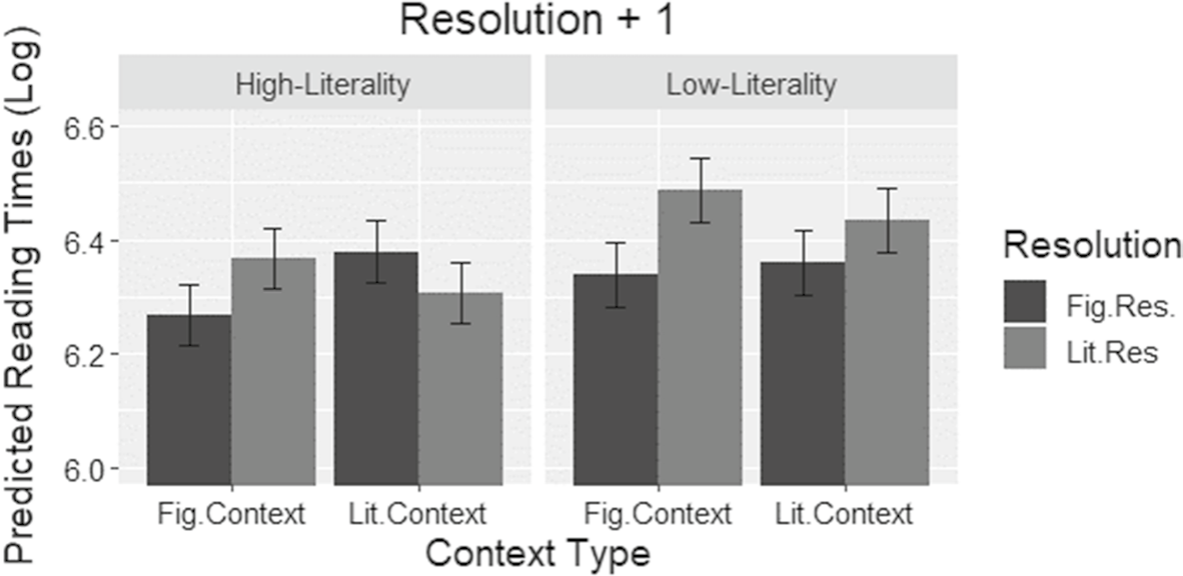
Log reading times predicted by the final model at Resolution + 1 Region divided into low- (left) and high-literality (right) idioms by biasing context. The black bars represent mean log reading times following a figurative resolution (Fig.Res.) and the grey bars following a literal resolution (Lit.Res.), all with standard error bars

The final model output for the *resolution *+* 1* region, derived following the same procedure as the other regions, is displayed in Table 5. The predicted log values from the final model can be seen in Fig. 3. Unlike in the other two regions, the text in this region was identical in all four conditions for each idiom. Main effects of *resolution type* (*β *= − 0.07, *t *= − 3.86, *p *< 0.001) and, marginally, *idiom literality* (*β *= − 0.07, *t *= − 1.84, *p *= 0.07) were present. Additionally, the effects of *region length* and *trial order* were generally in line with those found in the previous regions. In addition to main effects, several interactions were also significant, setting our data apart from earlier studies. Both *biasing context* by *resolution type* (*β *= − 0.11, *t *= − 3.23, *p *< .01) and *idiom literality* by *resolution type* (*β *= 0.10, *t *= 2.75, *p *< 0.01) were significant, and the three-way interaction of *biasing context* by *resolution type* by *idiom literality* was marginally significant (*β *= − 0.13, *t *= − 1.77, *p *= 0.07).

**Table 5:** Resolution + 1 region LMER model Output

Factor	*β* (SE)	*t*	*p*
Intercept	6.354 (.04)	142.065	2.00E−16***
Biasing context	− 0.021 (.01)	− 1.168	0.2431
Idiom literality	− 0.072 (.03)	− 1.842	0.0766.
Resolution type	− 0.070 (.01)	− 3.869	0.0001***
Region length	0.049 (.01)	2.945	0.0091**
Trial order	− 0.091 (.01)	− 7.561	1.89E−11***
Context × literality	− 0.031 (.03)	− 0.858	0.3911
Context × resolution-type	− 0.118 (.03)	− 3.238	0.0012**
Literality × resolution-type	0.100 (.03)	2.754	0.006**
Context × literality × resolution-type	− 0.130 (.07)	− 1.777	0.0759.

Random effects	Variance	SD
Subject	0.072	0.269
Idiom literality	0.017	0.131
Item	0.004	0.063

.*p *< 0.1; *p < .05; **p < .01; ***p < .001

In order to better interpret the interactions, we performed post hoc analyses using simple slopes on the relevant variables individually. First, the effect of *idiom literality* on *biasing context* as well as its involvement in the three-way interaction with *resolution type* was investigated by recoding *idiom literality* (first as high-literality = 0, low-literality = 1, subsequently reversed) and re-running the original model. Results showed that *resolution type* was significant in the case of low-literality idioms (*β *= − 0.12, *t *= − 4.44, *p *< 0.001) but not in high-literality idioms (*β *= − 0.02, *t *= − 0.82, *p *= 0.40). This confirms the pattern (see Fig. 3) in low-literality idioms that figurative resolutions were significantly better than literal ones. However, the results also show that the interaction between *biasing context* and *resolution type* was significant for the high-literality idioms (*β *= − 0.18, *t *= − 3.81, *p *< 0.001) and not for the low-literality idioms (*β *= − 0.05, *t *= − 0.97, *p *= 0.32).

In order to investigate the nature of this interaction in high-literality idioms, *biasing context* was also recoded (first as figurative context = 0, literal context = 1, subsequently reversed), and the original model was used again, keeping high-literality idioms as the intercept. The results display main effects of *resolution type* for both literal (*β *= 0.07, *t *= 2.04, *p *< 0.05) and figurative contexts (*β *= − 0.11, *t *= − 3.36, *p *< 0.001) in opposite directions. This finding suggests that, for high-literality idioms, congruently resolving endings were read more quickly than incongruently resolving endings (e.g., “The new schoolboy… wanted to *break the ice* with his peers…” and “The chilly Eskimo…wanted to *break the ice* on the lake…” vs. “The new schoolboy… wanted to *break the ice* on the lake …” and “The chilly Eskimo…wanted to *break the ice* with his peers …”). While this is a reflection of the marginally significant three-way interaction, these results confirm the trends displayed in the high-literality idioms (right) in Fig. 3.

In order to investigate any possible effects not already summarized by the recoding of *idiom literality* and then *biasing context *according to literality, *biasing context* was recoded (first as figurative context = 0, literal context = 1, subsequently reversed) and applied to the original model with no other changes. Where contexts were figurative, both the effects of *idiom literality* (*β *= − 0.08, *t *= − 2.03, *p *< 0.05) and *resolution type* (*β *= − 0.13, *t *= − 5.05, *p *< 0.001) were significant. This result suggests that figurative contexts cause significant benefits and costs where resolutions do not match these expectations and that figurative contexts improve readings for high-literality idioms more than low-literality idioms. In the case of literal contexts, these factors were involved in a two-way interaction between these factors (*β *= − 0.16, *t *= − 3.172, *p *< 0.01), which was already explored in the first post hoc analysis.

## Discussion and Conclusions

With this self-paced reading study, we aimed to further examine the activation and role of literal and figurative meaning during the time-course of idiomatic processing. Specifically, we asked how context impacts access to these interpretations and what limits the level of literality of an idiom can impose in both cases where contextual expectations are met and turn out to be false. By examining three regions of interest (the *idiom*, the *resolution*, and the *resolution *+* 1* regions), we were able to see effects of context and literality as both figurative and literal interpretations were integrated with contextual meaning. Our study fills some of the gaps in similar research conducted in the field and provides evidence that context has a limited role in meaning activation, and factors such as idiom literality can supersede standard processing and integration mechanisms under certain conditions. We will briefly discuss the implications of the reading times in the first two regions before focusing on the final region.

Notably, our findings show some critical differences from those of previous studies (e.g., Holsinger and Kaiser 2013) and also corroborate previous findings on meaning constitution that suggest a processing strategy that adapts to contextual and linguistic cues rather than unfolding in a uniform manner. We did not detect any early differences (i.e., *idiom* region) between idiom types based on context, which suggests similar processing during reading. The well-documented effect that idioms are read faster following a supporting context (e.g., Gibbs 1980; Holsinger and Kaiser 2013; Swinney and Cutler 1979) was not observed in our data. However, we presume that one reason this effect fails to reach significance could be due to the short length and unpredictability of the idioms in the present study (see e.g., Fanari et al. 2010) as well as to the possibility that effects which appear only very briefly (i.e., on single words) are lost in a phrase-by-phrase design.

Our findings in the *resolution* region, combined with the differences between this and the following region, suggest that as sentence comprehension unfolds, processing strategies may also be adaptive. The effect found in the *resolution* region in which there was a cost for figurative interpretations following literal contexts is in line with the conclusions drawn by Holsinger and Kaiser (2013). However, unlike the region in which this effect was found in their study, there is not yet evidence for the benefit of context in this region in our data, so conclusions based on this effect alone must be drawn with care. It may be evidence that early processing strategies indeed involve necessary literal composition, and for that reason figurative interpretations are costly where immediately unexpected. In this case, idiom processing necessarily involves literal processing to a certain extent. Theories of idiom processing that ignore these earlier processes or the possibility of literal processing at all (e.g., Gibbs 1980) are unsupported by this evidence. However, we do not propose that this suggests a literal processing priority. Rather, at this early stage in processing, prior to integration processes, the flexibility demonstrated in later regions is not yet evident at as literal processing may be most prominent or detectable at this point during processing. In order to best understand how such processing unfolds, this region must be considered as a predecessor to the final region, discussed below. Additionally, it should also be considered that this region draws comparisons between reading times of differing phrases.

Most importantly, the results in the final region (*resolution *+* 1*) are both in line with earlier studies (e.g., Canal et al. 2017; Rommers et al. 2013) and show key differences in reading time patterns suggesting that processing strategies may divide based on idiom-literality. Like Holsinger and Kaiser (2013), in our high-literality idioms, the greatest facilitation was found for sentences with a figurative bias and a figurative resolution. Figurative meanings did benefit the most from context for these idioms. But unlike their results, ours show that facilitation was still present for sentences with a literal bias and a literal resolution and that costs were greatest when literal endings followed figurative biasing contests. Our results are in line with those from both Canal et al. (2017) and Rommers et al. (2013), identifying literal contextual effects. These results also follow suit with Titone and Connine (1994b) concerning high-literality idioms, as both studies show evidence of literal meaning activation. Notably, the contexts in the current study were much longer than those used by Holsinger and Kaiser, and we expect that this may be one reason that effects not detected in the previous study were found in ours (see e.g., Ortony et al. 1978).

Another important result from the final region was that our low-literality idioms were not impacted by context. Rather, reading times were faster in the case that sentences resolved figuratively. These results were consistent with our predictions for this group of idioms, and generally with other studies examining low-literality idioms (e.g., Titone and Connine 1994b). While Titone and Connine (1994b) only found such a result with predictable, low-literality idioms, they only looked at automatic activation on the word-level. Thus, while this result provides a difference to this earlier study, the results are not contradictory. The results from this region also further impress upon differences in contextual effects found in the Holsinger and Kaiser (2013) study as well as the results found by Canal et al. (2017) and Rommers et al. (2013), in which literal contextual effects were identified as a whole.

Notably, the implications of these differences found in the *resolution* +*1* region imply that high- and low-literality idioms are not equally sensitive to contextual effects and may encourage differing processing strategies. In the case of high-literality idioms, processing may be more adaptive and take context into account. However, for low-literality idioms, this seems not to be the case; rather, a purely figurative processing strategy is preferred. Thus, these idioms provide evidence for one of the limitations of the impact of context on idiom processing. Unlike the resolution region itself, lexical items in this region were identical in all conditions and observed differences in reading times here can be attributed to the manipulations in our study.

Importantly, these results also impress upon the need for literal composition during idiom processing. The differences in effects between the two types of idioms suggest that, while literal processing does seem to be an important part of early processing mechanisms, in contrast with the claims of Holsinger and Kaiser (2013), literal processing may be abandoned or suppressed if either appropriate supporting context is present or an idiom has a low-literality. While we did see an early cost for unexpected figurative interpretations, this may reflect the process of late idiom recognition before processing becomes adaptive. In line with research from Rommers et al. (2013) and Canal et al. (2017), our low-literality idioms show evidence for abandonment of literal computation regardless of context. In addition to a lack of facilitation of literal resolutions compared to figurative resolutions, the general cost for all literal resolutions suggests that the conventional nature of these idioms might contribute to this processing strategy (i.e., experience with these idioms, used primarily figuratively, renders literal meanings unlikely). However, our lack of evidence for a contextual effect in this case is not necessarily evidence that it does not occur. Rather, the low literality of these idioms may simply be a stronger signal than context. For the high-literality idioms, the same process of abandonment of literal composition after recognition cannot entirely explain the data. Canal et al. (2017) found evidence that for high-literality, predictable idioms context mediated whether semantic analysis occurred. Their results also suggested that these effects occur very early in recognition. While our effects were found later than the effects discussed in their EEG data, typical of reading studies, it may be a case of shifting to a differentiated or even shallower processing of literal constituents (see also Peterson et al. 2001). This more subtle processing shift would be consistent with the lacking facilitation effect and heightened recovery costs in incongruent conditions. Following this pattern, it is feasible that the same effect may simply occur earlier or more prominently in low-literality idioms. In this case, context and literality could mediate when shallow processing becomes suppressed literal computation. Of course, given the nature of the task in the current study, it should also be noted that the late effects may also reflect evaluative processes in reading (e.g.,Rapp and Mensink 2011).

While the goal of our study was not to determine a model of processing for idioms, our results can be aligned with general research in the field already outlined. Unlike earlier studies, our data cannot speak to support an early advantage for literal interpretations, nor a purely figurative-first approach as they make uniform predictions about meaning retrieval and integration, and such models therefore cannot account for differences in context integration based on idiom literality. However, both hybrid models can account for the effects present in the final region, though, they do not explain the differences between idiom literality equally well. While the Configuration Hypothesis (Cacciari and Tabossi 1988) should support earlier or stronger recognition of low-literality idioms (see e.g., Titone and Connine 1994b), it is unclear about predictions of literal composition, and therefore silent on whether or not it is in line with the results seen for our high-literality idioms. A later (or weaker) recognition of high-literality idioms may account for the stronger use of context in processing, and therefore the facilitation effect evidenced by the advantage for matching biases and resolutions. The Hybrid Representation theory (Sprenger et al. 2006) can account for this behavior in a clearer manner. As this hypothesis allows for a stronger level of competition between the two interpretations, as both are activated during processing, the model seems to predict that idioms with a greater capacity to be interpreted literally should have more competition between meanings than those without (i.e., low-literality idioms). Thus, it can explain why literal interpretations are abandoned in low-literality idioms in all contextual situations, while context more readily impacts the competition between meanings in high-literality idioms.

Importantly, the current study has added to the body of literature that supports more heterogenous accounts of idioms and their processing, but it faces limitations in its claims about exactly what causes these differences in processing strategies. Here, literality, as defined by the potential for literal interpretation and examined as a binary variable, was shown to interact with context in idiomatic processing in a critical manner: literality may cause processing to proceed either more or less flexibly (concerning high-literality and low-literality idioms respectively). However, as discussed briefly in the introduction, this factor overlaps critically with other idiomatic properties such as ambiguity (e.g., Cacciari and Tabossi 1988), saliency (e.g., Giora 1997), and even meaning dominance (e.g., Milburn and Warren 2019), and it is unclear precisely which of and to what extent these factors may have impacted the results of the current study. Although high- or low-literality idioms can greatly overlap with ambiguous and non-ambiguous idioms respectively, these two terms differ from one another in that literality accounts only for the potential of a literal interpretation; ambiguity should also account for the likelihood of such an interpretation, as an idiom is only ambiguous if both interpretations are actually likely. This likelihood for ambiguity is also complicated by subjective familiarity with an idiom’s figurative and literal uses as well as the dominance of such uses (e.g., Cronk et al. 1993; Milburn and Warren 2019), all factors which contribute to the salience of a particular meaning (see e.g., Giora 1997). Though some idioms may have a high-literality, or have both plausible literal and figurative interpretations, these two interpretations may not be equally salient for users. In light of the differences between the two types of idioms examined in this study, it is also conceivable that similar results may be achieved when looking at ambiguity, saliency, or even meaning dominance as predicting factors, since low-literality idioms seem to also critically differ from high-literality idioms among these properties (i.e., low-literality idioms are typically less ambiguous; may be more salient in the figurative meaning; and are more often used in the figurative sense, as reflected in our norming studies). While the current study was not designed to tease apart these properties, future studies should look at a broader spectrum of such properties and may even employ them as a scale rather than a binary factor in order to better do so.

Overall, our findings fill in some of the gaps in current research on idiom processing, specifically in addressing questions of figurative and literal meaning constitution on a phrasal level. Our data support a processing strategy that is sensitive to context, but in which these contextual effects are mediated by idiom literality. We conclude that processing follows a single, adaptive pattern until a threshold of information can be reached that suppresses or qualitatively changes the nature of literal computation. This threshold can be mediated by context, pushing more ambiguous, high-literality idioms across this threshold only when context also supports such a strategy, and by literality, which can supersede context in strength if low-literality idioms cause the threshold to be reached earlier, for example. Future studies are still needed to provide more evidence on what happens earlier in processing concerning context and literality, among other properties, and what additional factors can impact a change in processing strategies.

## Appendix

**Table:**

	Subject	Context 1	Context 2	Pre-idiom	Idiom	Resolution	Resolution + 1	Wrap-up
1a	The new schoolboy,	who didn’t know	anyone in his class,	just wanted to	break the ice	with his peers	as soon as possible	monday morning.
1b	The new schoolboy,	who didn’t know	anyone in his class,	just wanted to	break the ice	on the lake	as soon as possible	monday morning.
1c	The cold Eskimo,	who was eager	to catch some fish,	just wanted to	break the ice	on the lake	as soon as possible	monday morning.
1d	The cold Eskimo,	who was eager	to catch some fish,	just wanted to	break the ice	with his peers	as soon as possible	monday morning.
2a	The fearless climber,	who was on a climb	alone in the mountains,	was ready to	play with fire	with any risk	if necessary	later on.
2b	The fearless climber,	who was on a climb	alone in the mountains,	was ready to	play with fire	from the grill	if necessary	later on.
2c	The young camper,	who was already bored	without any of his friends,	was ready to	play with fire	from the grill	if necessary	later on.
2d	The young camper,	who was already bored	without any of his friends,	was ready to	play with fire	with any risk	if necessary	later on.
3a	The trained therapist,	who just started	with a new patient,	really hoped to	scratch the surface	of the problem	without any delay	at all.
3b	The trained therapist,	who just started	with a new patient,	really hoped to	scratch the surface	off the ticket	without any delay	at all.
3c	The gambling addict,	who just wanted	to win the grand prize,	really hoped to	scratch the surface	off the ticket	without any delay	at all.
3d	the gambling addict,	who just wanted	to win the grand prize,	really hoped to	scratch the surface	of the problem	without any delay	at all.
4a	The new model,	who tried her best	to wear the latest fashion designs,	had chosen to	follow the crowd	with this trend	at her own risk	that day.
4b	The new model,	who tried her best	to wear the latest fashion designs,	had chosen to	follow the crowd	through the city	at her own risk	that day.
4c	The Italian tourist,	who was easily lost	when traveling to new places,	had chosen to	follow the crowd	through the city	at her own risk	that day.
4d	The Italian tourist,	who was easily lost	when traveling to new places,	had chosen to	follow the crowd	with this trend	at her own risk	that day.
5a	A tired housekeeper,	who just wanted	to go home and rest,	wasn’t willing to	lift a finger	by offering help	even when asked	to do so.
5b	A tired housekeeper,	who just wanted	to go home and rest,	wasn’t willing to	lift a finger	off the keys	even when asked	to do so.
5c	The office secretary,	who was always typing	during important meetings,	wasn’t willing to	lift a finger	off the keys	even when asked	to do so.
5d	The office secretary,	who was always typing	during important meetings,	wasn’t willing to	lift a finger	by offering help	even when asked	to do so.
6a	A bride-to-be,	who spent the night	thinking about her future,	hoped not to	have cold feet	about the wedding	the next morning	before breakfast.
6b	A bride-to-be,	who spent the night	thinking about her future,	hoped not to	have cold feet	from the snow	the next morning	before breakfast.
6c	The young hiker,	who spent the night	without fire in the woods,	hoped not to	have cold feet	from the snow	the next morning	before breakfast.
6d	The young hiker,	who spent the night	without fire in the woods,	hoped not to	have cold feet	about the wedding	the next morning	before breakfast.
7a	The popular teenager,	who liked to gossip	with his group of friends,	had managed to	spill the beans	about the surprise	even faster	than expected.
7b	The popular teenager,	who liked to gossip	with his group of friends,	had managed to	spill the beans	on the stove	even faster	than expected.
7c	The assistant chef,	who didn’t seem	to do anything well,	had managed to	spill the beans	on the stove	even faster	than expected.
7d	The assistant chef,	who didn’t seem	to do anything well,	had managed to	spill the beans	about the surprise	even faster	than expected.
8a	The working mother,	who always dreamed	of a long-lasting career,	was planning to	wear the pants	in the family	to the surprise	of many.
8b	The working mother,	who always dreamed	of a long-lasting career,	was planning to	wear the pants	with the pink dots	to the surprise	of many.
8c	The aspiring dancer,	who was worried	about her audition outfit,	was planning to	wear the pants	with the pink dots	to the surprise	of many.
8d	The aspiring dancer,	who was worried	about her audition outfit,	was planning to	wear the pants	in the family	to the surprise	of many.
9a	The financial manager,	who had to work	with a small budget,	would attempt to	pull the plug	on the project	after the discussion	that afternoon.
9b	The financial manager,	who had to work	with a small budget,	would attempt to	pull the plug	from the drain	after the discussion	that afternoon.
9c	The local plumber,	who was ready	to get right to work,	would attempt to	pull the plug	from the drain	after the discussion	that afternoon.
9d	The local plumber,	who was ready	to get right to work,	would attempt to	pull the plug	on the project	after the discussion	that afternoon.
10a	The gay couple,	who had been in love	for many years,	was able to	tie the knot	in a church	after much time	and effort.
10b	The gay couple,	who had been in love	for many years,	was able to	tie the knot	in the yarn	after much time	and effort.
10c	The expert knitter,	who was concentrated	on her newest scarf,	was able to	tie the knot	in the yarn	after much time	and effort.
10d	The expert knitter,	who was concentrated	on her newest scarf,	was able to	tie the knot	in a church	after much time	and effort.
11a	The substitute teacher,	who was in great need	of a break from the class,	was ready to	draw the line	at one interruption	right away	the next morning.
11b	The substitute teacher,	who was in great need	of a break from the class,	was ready to	draw the line	on the page	right away	the next morning.
11c	The new architect,	who had been sketching	the same picture for days,	was ready to	draw the line	on the page	right away	the next morning.
11d	The new architect,	who had been sketching	the same picture for days,	was ready to	draw the line	at one interruption	right away	the next morning.
12a	The teenage girl,	who was always speaking	without thinking,	just wanted to	eat her words	from the fight	late last night	in her room.
12b	The teenage girl,	who was always speaking	without thinking,	just wanted to	eat her words	from her spoon	late last night	in her room.
12c	The hungry student,	who was distractedly playing	with her alphabet soup,	just wanted to	eat her words	from her spoon	late last night	in her room.
12d	The hungry student,	who was distractedly playing	with her alphabet soup,	just wanted to	eat her words	from the fight	late last night	in her room.
13a	The freelance writer,	who often started	political debates,	didn’t want to	lose his cool	out of anger	so quickly	that morning.
13b	The freelance writer,	who often started	political debates,	didn’t want to	lose his cool	from the shade	so quickly	that morning.
13c	The sweaty runner,	who was recovering	under a tree,	didn’t want to	lose his cool	from the shade	so quickly	that morning.
13d	The sweaty runner,	who was recovering	under a tree,	didn’t want to	lose his cool	out of anger	so quickly	that morning.
14a	The young girl,	whose close friend	was openly upset,	would offer to	lend an ear	with some advice	as a gesture	of kindness.
14b	The young girl,	whose close friend	was openly upset,	would offer to	lend an ear	from her doll	as a gesture	of kindness.
14c	A little girl,	whose brother rebuilt	figurines from broken ones,	would offer to	lend an ear	from her doll	as a gesture	of kindness.
14d	A little girl,	whose brother rebuilt	figurines from broken ones,	would offer to	lend an ear	with some advice	as a gesture	of kindness.
15a	The jazz musician,	who had been trying	to get a record deal,	was willing to	give the world	for some fame	if it would help	his cause.
15b	The jazz musician,	who had been trying	to get a record deal,	was willing to	give the world	a new name	if it would help	his cause.
15c	The retired astronaut,	who had been tracking	a new planet in space,	was willing to	give the world	a new name	if it would help	his cause.
15d	The retired astronaut,	who had been tracking	a new planet in space,	was willing to	give the world	for some fame	if it would help	his cause.
16a	A car dealer,	who was able to recognize	customers’ tastes in vehicles,	would try to	stretch a point	in any sales pitch	if necessary	for success.
16b	A car dealer,	who was able to recognize	customers’ tastes in vehicles,	would try to	stetch a point	into a line	if necessary	for success.
16c	A sketch artist,	who was up late designing	her newest drawing,	would try to	stretch a point	into a line	if necessary	for success.
16d	A sketch artist,	who was up late designing	her newest drawing,	would try to	stretch a point	in any sales pitch	if necessary	for success.
17a	A young woman,	who had been fighting	with her boyfriend,	had decided to	face the music	with an apology	late last night	at the event.
17b	A young woman,	who had been fighting	with her boyfriend,	had decided to	face the music	from the speakers	late last night	at the event.
17c	The lead singer,	who had been struggling	with hearing problems,	had decided to	face the music	from the speakers	late last night	at the event.
17d	The lead singer,	who had been struggling	with hearing problems,	had decided to	face the music	with an apology	late last night	at the event.
18a	The cool kid,	who could pick	any date to the dance,	had decided to	play the field	instead of choosing	this time around	as expected.
18b	The cool kid,	who could pick	any date to the dance,	had decided to	play the field	behind the school	this time around	as expected.
18c	The soccer player,	whose team always	needed new challenges,	had decided to	play the field	behind the school	this time around	as expected.
18d	The soccer player,	whose team always	needed new challenges,	had decided to	play the field	instead of choosing	this time around	as expected.
19a	The crop farmer,	who was struggling	with a long drought,	wasn’t able to	turn the tide	of his luck	as he hoped	he could.
19b	The crop farmer,	who was struggling	with a long drought,	wasn’t able to	turn the tide	of the water	as he hoped	he could.
19c	The deep-sea diver,	who was swimming	much later than expected,	wasn’t able to	turn the tide	of the water	as he hoped	he could.
19d	The deep-sea diver,	who was swimming	much later than expected,	wasn’t able to	turn the tide	of his luck	as he hoped	he could.
20a	The famous songwriter,	who always used	his emotions in his songs,	wasn’t hoping to	have the blues	from a heartbreak	when getting started	on the project.
20b	The famous songwriter,	who always used	his emotions in his songs,	wasn’t hoping to	have the blues	on his paintbrush	when getting started	on the project.
20c	The art student,	who was often lazy	about cleaning his art supplies,	wasn’t hoping to	have the blues	on his paintbrush	when getting started	on the project.
20d	The art student,	who was often lazy	about cleaning his art supplies,	wasn’t hoping to	have the blues	from a heartbreak	when getting started	on the project.
21a	The powerful politician,	who was always	covering up a new scandal,	was going to	hit the headlines	of every newspaper	before going to work	Thursday morning.
21b	The powerful politician,	who was always	covering up a new scandal,	was going to	hit the headlines	with his fist	before going to work	Thursday morning.
21c	The old man,	who was always	upset while reading politics,	was going to	hit the headlines	with his fist	before going to work	Thursday morning.
21d	The old man,	who was always	upset while reading politics,	was going to	hit the headlines	of every newspaper	before going to work	Thursday morning.
22a	The new manager,	who didn’t have	much working experience,	really needed to	fit the bill	for the job	without any delay	at work.
22b	The new manager,	who didn’t have	much working experience,	really needed to	fit the bill	in the folder	without any delay	at work.
22c	The nervous waiter,	who was worried	about his observant boss,	really needed to	fit the bill	in the folder	without any delay	at work.
22d	The nervous waiter,	who was worried	about his observant boss,	really needed to	fit the bill	for the job	without any delay	at work.

All experimental items. Items 1–11 are high-literality idioms, and items 12–22 are low-literality idioms. For each example, (a) has a figuratively biasing context and a figurative resolution, (b) has a figuratively biasing context and a literal resolution, (c) has a literally biasing context and a literal resolution, and (d) has a literally biasing context and a figurative resolution
